# Fatal case of co-infection with dengue virus and *Neisseria meningitidis* during a dengue epidemic in the state of Rio de Janeiro, Brazil

**DOI:** 10.1099/jmmcr.0.005055

**Published:** 2016-08-30

**Authors:** Ivano de Filippis, Priscila Conrado Guerra Nunes, Claudia Ferreira de Andrade, Bianca de Santis Gonçalves, Eliane Saraiva de Araújo, Itacirema de Oliveira Bezerra, Ivan Rocha Ferreira da Silva, Rita Maria Nogueira, Ana Maria Bispo de Filippis

**Affiliations:** ^1^​ National Institute for Quality Control of Health - INCQS, FIOCRUZ, Rio de Janeiro, Brazil; ^2^​ Flavivirus Laboratory, Oswaldo Cruz Institution, FIOCRUZ, Rio de Janeiro, Brazil; ^3^​ State Secretary of Health, Rio de Janeiro, Brazil; ^4^​ Evandro Chagas National Institute of Infectious Diseases, FIOCRUZ, Rio de Janeiro, Brazil

**Keywords:** Dengue, meningococcal disease, diagnostic, PCR, RT-PCR

## Abstract

**Introduction:**

Dengue and meningococcal disease are caused by two different agents: a flavivirus and a Gram-negative bacterium, respectively. The first symptoms of both diseases can be indistinct and a rapid and accurate diagnosis is crucial, considering that both diseases are associated with high morbidity and mortality, representing a major public-health problem in Brazil.

**Case presentation:**

We report a fatal case of co-infection of dengue virus (DENV) and *Neisseria meningitidis* in a 54-year-old patient. The serum tested positive for DENV NS1 antigen, and *N. meningitidis* serogroup C was detected by *nspA*-PCR. Following the initial positive result for DENV infection, rRT-PCRwas performed and DENV-4 was confirmed.

**Conclusion:**

Our report highlights the importance of accurate differential diagnosis during periods of high circulation of DENV, in order to provide adequate management and an improved outcome.

## Introduction

Despite being aetiologically distinct diseases, both dengue, caused by one of four serotypes of dengue virus (DENV), and meningococcal meningitis, caused by the Gram-negative bacterium *Neisseria meningitidis*, can initially present with similar clinical symptoms, such as high fever, dizziness (potentially evolving to central nervous system injuries), petechial rash or an occasionally fatal shock syndrome (Coureuil *et al*., 2013; Stephens *et al*., 2007).

Dengue is hyperendemic in Brazil, with outbreaks occurring almost yearly. The co-circulation of all four DENV serotypes in all Brazilian states has increased the incidence of severe cases, hospitalizations and death secondary to dengue infection (Siqueira *et al*, 2005). Since the first reports of dengue in the 1980s, more than 10 million cases have been diagnosed (Brasil Ministério da Saúde SVS/MS, 2016). During 2012, 565 510 cases of dengue infection were reported in Brazil, including 4055 cases of severe dengue infection and 284 related deaths (Brasil Ministério da Saúde SVS/MS, 2016; van Panhuis *et al*., 2014; Zambrano & San Martin, 2014; Pan American Health Organization (PAHO) 2016, PAHO/World Health Organization programme – dengue, http://www.paho.org/hq/index.php?option=com_topics&view=article&id=1&Itemid=40734, accessed on 25 May 2016. In the state of Rio de Janeiro, a total of 181 169 cases with 43 deaths were reported in 2012. In the same year, a total of 2083 cases of meningococcal disease were notified, with 440 deaths in Brazil. At the same time in Rio de Janeiro, a total of 390 cases of meningococcal disease with 96 deaths were reported (SVS/SES-RJ, 2016).

Unlike dengue, almost all pathogenic *N*. *meningitidis* serogroups are preventable by immunization; however, meningococcal disease is still endemic in our country, being the leading cause of bacterial meningitis in Brazil. The incidence of meningococcal disease in Brazil ranges from 1–1.5 cases per 100 000 inhabitants, with a reported case-fatality rate over the past 10 years of about 11 % when presenting as meningitis, 16 % as meningitis with septicaemia, rising to 38 % in septicaemia, with an overall case-fatality rate of 20 % (Brasil Ministério da Saúde SVS/MS, 2015). The clinical forms of meningitis, meningitis with septicaemia and septicaemia alone accounted for 39.2, 33 and 27.8 %, respectively, of the confirmed cases of meningococcal disease in Brazil during this period of time. (Gorla *et al*., 2011). Meningococcal disease is frequently observed in epidemic waves or in local outbreaks, which might be influenced by virulent strains, crowded locations with poor ventilation, climate and lower socioeconomic status. Some authors have previously described bacterial co-infection during the course of a viral infection with DENV (Srirangaraj *et al*., 2014; Pérez Rodríguez *et al*., 2014; Tsai *et al*., 2013; Nagassar *et al*., 2012); however, this is, to the best of our knowledge, the first report of a fatal case of co-infection of DENV and *N. meningitidis*.

Patients presenting with DENV or with *N*. *meningitidis* infection might experience similar symptoms, including high fever, prostration, joint pain, neck stiffness and petechial rash. With regards to the potentially overlapping signs and symptoms, rapid accurate diagnosis is vital, to aid proper management and improve the outcome.

## Case report

During a dengue outbreak in 2013 in the state of Rio de Janeiro, a 54-year-old gentleman presented to an urgent care facility with fever and dyspnoea. Initially, the diagnosis of community acquired pneumonia was made and the patient was discharged home with a prescription for a 10 day course of oral amoxicillin with clavulanic acid, 500 mg every 8 h. Three days later, he was readmitted with severe headache, fever and confusion, with peripheral cyanosis and a petechial rash. On laboratory testing, the patient was found to be thrombocytopenic and had acute renal failure, requiring urgent haemodialysis. Due to the presenting clinical picture, blood samples were collected to test for dengue and meningococcal infection. Blood cultures were negative for *N. meningitidis.* A lumbar puncture with cerebral spinal fluid analysis disclosed an opening pressure of 30 cm H_2_O, a cell count of 750 white blood cells per field (90 % neutrophils), protein levels of 102 mg dl^−1^ and glucose levels of 45 mg dl^−1^. Cerebral spinal fluid cultures were not performed by the admitting hospital. The patient was immediately started on ceftriaxone, 2 ***g*** intravenously every 12 h. The results of NS1 antigen testing and RT-qPCR for dengue (Lanciotti *et al*., 1992) were positive for DENV-4 and anti-dengue IgM was negative. A *nspA*-PCR assay (de Filippis *et al*., 2005) performed to detect meningococcal disease confirmed *N. meningitidis *infection, and serogroup C was determined by *siadA*-PCR (Tzanakaki *et al*., 2003). The amplified *nspA *DNA fragment was sequenced in both directions and confirmed as the *nspA* gene. The DNA sequence was submitted to GenBank under accession number KP721282. Unfortunately, after 5 days in a critical care unit, the patient developed multi-system organ failure and died. The medical team in charge reported meningococcal disease as the cause of death on his death certificate.

## Discussion

The natural habitat of* N. meningitidis* is the human nasopharynx, where it can live as a commensal in about 10 % of the population for a few months without causing invasive disease. These individuals are called asymptomatic carriers. For unknown reasons, 5–10 % of the carriers might develop invasive disease with devastating consequences, such as meningitis, sepsis (meningococcemia) and acute adrenal failure (Waterhouse–Friederiksen’s syndrome) (Gasparini *et al*., 2012; Varon *et al*., 1998). Meningococcal disease is a vaccine-preventable disease for 4 of the 5 most common serogroups (A, C, W135, Y). At the time of the reported case, there was no vaccine available against serogroup B meningococci. In the beginning of 2015, a four-component protein vaccine against serogroup B was introduced in immunization calendars in Europe and the USA. In the same year, the vaccine was also approved in Brazil for use in private vaccination clinics. The world’s first dengue vaccine was released in 2015 and was approved for use in Brazil in January 2016, but it is not yet listed in the national immunization calendar. Dengue disease has no specific antiviral treatment currently available.

It is known that dengue affects the immune system, predisposing patients to other infections. It is possible that the patient was initially an asymptomatic carrier of the bacterium, who afterwards acquired DENV infection, and the resulting low immunity caused by dengue led to meningococcal invasive disease. Conversely, the most important human defence against bacterial infection is the immune system, so it is also possible that the patient was acutely exposed to *N*. *meningitidis* while his immune defences where compromised by an acute DENV infection.

Supportive care is basically the only therapy against dengue. Management of severe dengue requires careful attention to fluid management and diligent treatment of haemorrhages. In addition, meningococcal disease is a preventable infection, and early administration of appropriate antibiotics can result in a successful outcome. There are several diseases that can present with strikingly similar symptoms, such as dengue, yellow fever, leptospirosis and meningococcal meningitis, among others. Therefore, it is crucial that even during periods of high circulation of dengue, cases with atypical clinical presentations, particularly the ones with neurological involvement, are further investigated for a potential co-infection with other agents. Different therapies are currently recommend for these diseases, and prompt medical attention with rapid and accurate diagnosis is imperative, as it might significantly impact the final outcome.
